# Intervening along the spectrum of tuberculosis: meeting report from the World TB Day nanosymposium in the Institute of Infectious Disease and Molecular Medicine at the University of Cape Town

**DOI:** 10.12688/gatesopenres.13035.4

**Published:** 2020-05-28

**Authors:** Sabelo Hadebe, Melissa Chengalroyen, Reto Guler, Kehilwe Nakedi, Anastasia Koch, Mohau Makatsa, Muki Shey, Suraj P. Parihar, Bryan Bryson, Mohlopheni J. Marakalala, Hlumani Ndlovu

**Affiliations:** 1 Division of Immunology and South African Medical Research Council (SAMRC) Immunology of Infectious Diseases, Department of Pathology, Faculty of Health Sciences, Institute of Infectious Diseases and Molecular Medicine (IDM), Cape Town, Westen Cape, 7925, South Africa; 2 SAMRC/NHLS/UCT Molecular Mycobacteriology Research Unit, Molecular Mycobacteriology unit, Division of Medical Microbiology, Department of Pathology, Faculty of Health Sciences, Institute of Infectious Disease and Molecular Medicine based (IDM), University of Cape Town, Cape Town, Western Cape, 7925, South Africa; 3 Department of Pathology, Faculty of Health Sciences, University of Cape Town, International Centre for Genetic Engineering and Biotechnology (ICGEB), Cape Town Component, Cape Town, Westen Cape, 7925, South Africa; 4 Wellcome Centre for Infectious Diseases Research in Africa (CIDRI-Africa), Institute of Infectious Diseases and Molecular Medicine (IDM) & Department of Medicine, Faculty of Health Sciences, University of Cape Town, Cape Town, Western Cape, 7925, South Africa; 5 Division of Chemical and Systems Biology, Department of Integrative Biomedical Sciences, Faculty of Health Sciences, Institute of Infectious Diseases and Molecular Medicine, University of Cape Town, Cape Town, Western Cape, 7925, South Africa; 6 Division of Medical Virology, Department of Pathology, Faculty of Health Sciences, Institute of Infectious Diseases and Molecular Medicine, University of Cape Town, Cape Town, Westen Cape, 7925, South Africa; 7 MIT Biological Engineering, Ragon Institute of MGH, MIT and Harvard, Cambridge, Massachusetts, MA 02142, USA; 8 Africa Health Research Institute, South Africa, Durban, KwaZulu Natal, South Africa; 9 Division of Infection and Immunity, University College London, London, South Africa

**Keywords:** Tuberculosis, TB/HIV co-infections, Host directed Therapies, transmission, new tools

## Abstract

Tuberculosis (TB), caused by the highly infectious *Mycobacterium* *tuberculosis*, remains a leading cause of death worldwide, with an estimated 1.6 million associated deaths reported in 2017. In South Africa, an estimated 322,000 (range 230,000-428,000) people were infected with TB in 2017, and a quarter of them lost their lives due to the disease. Bacille Calmette-Guérin (BCG) remains the only effective vaccine against disseminated TB, but its inability to confer complete protection against pulmonary TB in adolescents and adults calls for an urgent need to develop new and better vaccines. There is also a need to identify markers of disease protection and develop novel drugs. It is within this backdrop that we convened a nanosymposium at the Institute of Infectious Disease and Molecular Medicine at the University of Cape Town to commemorate World TB Day and showcase recent findings generated by early career scientists in the institute. The speakers spoke on four broad topics: identification of novel drug targets, development of host-directed drug therapies, transmission of TB and immunology of TB/HIV co-infections.

## Disclaimer

The views expressed in this article are those of the author(s). Publication in Gates Open Research does not imply endorsement by the Gates Foundation.

## 

Tuberculosis is a debilitating communicable disease affecting millions of people worldwide, with the highest incidence and mortality rates in Southern Africa. In 2017 an estimated 1.6 million people died of TB worldwide, making it the leading cause of death due to a single infectious agent ^1^. TB is transmitted through airborne inhalation of *Mycobacterium tuberculosis* (Mtb) that seeds in alveolar spaces in the lungs. Realizing the limitations of the TST and IGRA, it has been estimated that almost one-third of the world population that is exposed to Mtb infection is able to control the infection and remain asymptomatic, otherwise known as latently infected ^2^. However, 5–10% of these latently infected individuals progress to active TB and manifest symptoms of the disease ^3^. More recently, the situation has been made more complex by the emergence of drug-resistant bacteria and co-infections with the human immunodeficiency virus (HIV). In 2017, the WHO estimated that 558 000 (range 483,000–639,000) people developed resistance to rifampicin (RR-TB), and 82% of these people had multi-drug resistant TB (MDR-TB) ^4^. This underpins an urgent need to develop better and efficacious vaccines than the currently available BCG and to develop novel drugs for TB.

## Identifying new drug targets

Mtb has a sophisticated metabolic repertoire: it is able generate its own nutrients, but it can also scavenge for some nutrients from the host ^5^. Studies have shown that Mtb is able to synthesise essential amino acids such as L-arginine and tryptophan, where deletion of these key metabolites restricts Mtb growth in culture and renders it more susceptible to host immune pressure reducing its survival ^6, 7^. Dr Melissa Chengalroyen, a research officer in the Molecular Mycobacteriology Research Unit, under the directorship/supervision of Prof Valerie Mizrahi, opened the morning session and spoke about the key elements required for Mtb survival and how Mtb sequesters these elements to evade host-recognition by non-conventional T cells. She then described a complex *de novo* riboflavin metabolic pathway and different tools to create Mtb mutants lacking key enzymes involved in this pathway to facilitate understanding of each step in the pathway. Her study showed that not all the enzymes involved in the riboflavin pathway are essential—some play redundant roles, while others are absolutely necessary for Mtb survival, and these may hold promise as new candidate drug targets for TB or play an essential role in alerting non-restricted T cell immune arm for faster clearance of the bacteria.

The second speaker in the morning session was Dr Kehilwe Nakedi, a postdoctoral research fellow in the laboratory of Prof Jonathan Blackburn. Her research project was aimed at identifying novel substrates for mycobacterial protein kinase G (PknG) using a mass spectrometry-based phosphoproteomics approach to elucidate mechanisms by which mycobacteria interfere with the host signalling during LTBI. She identified 3164 phosphopeptides with high confidence using label-free data analysis ^8^. Moreover, she identified 63 host phosphopeptides that were phosphorylated in macrophages infected with *M. bovis* BCG only and not those infected with the mutant lacking PknG. Further analysis of the data revealed that these substrates phosphorylated in the presence of PknG play a key role in regulating actin polymerisation and cytoskeleton integrity ^8^. This work suggest that pathogenic mycobacteria survive inside the host macrophages during early TB infection through interfering with the host’s cytoskeletal dynamics mediated by PknG.

## Development of host-directed drug therapies

Although the currently available TB treatment regimens are effective at killing the bacteria, the emergence of drug resistance and the long duration of treatment threaten their long-term efficacy. This underpins an urgent need to develop new anti-TB drugs and exploration of other treatment strategies to control the disease. One such strategy is to develop host-directed drug therapies (HDTs) with the aim of boosting the host’s innate ability to fight the infection and also limit the deleterious tissue pathology ^9^. Although this field is still in its infancy, it holds huge potential as adjunctive therapy for TB in clinical settings with a high disease burden.

Associate Prof Reto Guler discussed in detail their published pre-clinical data on the use of statins as a potential host-directed therapy for TB in mice ^10, 11^. He then spoke about the translation of this work in a proof-of-concept phase IIB, double-blind, randomized, placebo-controlled trial launched in Khayelitsha township and funded by the European & Developing Countries Clinical Trials Partnership ((EDCTP), RIA2017T-2004). The coordinator of this consortium is Reto Guler (University of Cape Town, Division of Immunology). Chief principal investigator of the clinical trial is Friedrich Thienemann (University of Zürich), local PI in Cape Town is Sandra Mukasa (University of Cape Town). Other project partners include Robert J. Wilkinson (Imperial College London), Claudia Schacht (LINQ Management GmbH, Germany), Gunar Günther and Emmanuel Nepolo (University of Namibia). The aim of the clinical trial is to investigate the use of statins to prevent chronic lung inflammation and potentially TB relapse in patients post completion of a standard TB treatment regimen. He also talked about other potential candidate targets for HDTs such as the transcriptional factor BATF2 ^12^ and microRNA-143 and microRNA-365 ^13^.

Another speaker on this topic was Dr Suraj Parihar, a Senior Research Officer and contributing investigator at CIDRI-Africa in the Institute of Infectious Diseases and Molecular Medicine (IDM). His talk focused mainly on preclinical studies that investigated the efficacy of repurposed drug (barberine) generally used to reduce blood glucose and cholesterol as HDTs for TB. He found that this drug was able to reduce lung inflammation in pre-clinical *in vitro* and *in vivo* models of TB. He then went on to speak about how growth factors can also be repurposed to enhance the killing ability of human and mouse macrophages. Although some of these studies are still at the pre-clinical stage, they hold a great promise and may pave way for alternative therapies and new clinical trials supported by strong pre-clinical data.

## Transmission of Mtb

Mtb is transmitted through the inhalation of Mtb-containing aerosols from person to person. Transmission of the bacteria is high in places such as schools, churches, mines, hospitals and heavily congested neighbourhoods of Cape Town such as informal settlements and townships ^14, 15^. In places with high prevalence of HIV, such as Khayelitsha, the rate of new Mtb infections accounts for more than half of TB cases. In 2006, it was reported that the incidence was as high as 1500/100,000 in some townships, exceeding that of national average ^16^. There are many challenges when it comes to measuring Mtb transmission via aerosol such as the low numbers of bacilli that can be captured, contamination by other bacterial or fungal particles in patients and other airborne particulate matter ^17^. New aerosol methods for capturing Mtb such as the respiratory aerosol sampling chamber (RASC) hold promise in quantifying rate of transmission especially in high endemic areas. The capacity to measure the rate of transmission and the type of bacilli strain circulating becomes even more critical in the era of high antimicrobial resistance ^18^.

Dr Anastasia Koch, a Carnegie Developing the Next African Leaders (DEAL) early career fellow, mentored by Prof Helen Cox and Prof Digby Warner, started off her talk by mentioning the potential of WGS technology in identifying Mtb genotypes resistant to the first line TB drugs ^19^. She discussed the major differences in genetic diversity observed in broth cultured Mtb populations and those derived directly from sputum, and moreover how simple culturing could result in loss of some of key genotypes. Anastasia then moved on to discussing the importance of getting a sample as close to that being transmitted by an infected person as possible in order to accurately study the strains that are being transmitted and driving disease in a community. She gave an example of how colleagues from the MMRU and the Desmond Tutu HIV Centre, have been able to capture and isolate Mtb strains from RASC bio-aerosols. She was able to culture these samples and compare whole genome of those strains with sputum induced strains. The ability to isolate Mtb from bio-aerosols and combining that with whole genome sequencing could greatly inform our understanding of TB transmission and treatment, particularly of emerging drug or multi-drug resistant strains.

## TB/HIV co-infection

More than 36.7 million people live with HIV/AIDS globally and most of these people live in sub-Saharan Africa. In 2017, it was estimated that more than 350 000 people died due to HIV/TB co-infection, making the TB the highest contributor to death in people living with HIV ^20^. HIV targets and depletes CD4 T cells at later stages of disease, including protective TB specific CD4 T cells ^21, 22^. Early antiretroviral (ARV) drug treatment is associated with improved outcomes and helps restore CD4 T cell count, including protective TB-specific CD4 T cells. However, there are complications associated with early ARV treatment in people who are also starting TB treatment. Some of these people develop TB-associated immune reconstitution syndrome (TB-IRIS), which can be fatal, unless controlled by host-directed immune suppressants such as corticosteroids ^23^.

To set the scene, Mohau Makatsa, a PhD candidate in the laboratory of Prof Wendy Burgers in the Division of Medical Virology, talked about a particular subset of CD4 T helper cells expressing IL-22, or “Th22 cells”, which are targeted by HIV and are implicated as a key player in natural resistance to TB ^24^. He showed how these cells can be stimulated *ex-vivo* by Mtb antigens and express different surface molecules compared to Th1 cells. There is a similar magnitude of these cells compared to Th1 cells in people with latent TB, but they are depleted in the peripheral blood in active TB disease and HIV co-infection. IL-22 has been shown to be important for the control of Mtb in mice ^25^. It is unclear how these cells play a role in the pathogenesis of TB in humans.

Dr Muki Shey, a Senior Research Officer & Wellcome Intermediate Fellow at CIDRI-Africa in the IDM followed and talked about identification of predictive immunological biomarkers associated with mortality in people who died of severe HIV-associated TB (HIV-TB). The study identified molecules such as interleukin-1 receptor agonist (IL-1Ra), IL-6, IL-8, macrophage inflammatory protein-1 beta (MIP-1B), and interferon gamma induced-protein 10 (IP-10) to be immune mediators that segregated patients who died from those who survived ^26^. The identification of these predictive markers could lead to better management of patients in the clinics, and also potentially lead to development of host-directed therapies.

## Engineering tools for TB

The international guest speaker at the Nanosymposium was Assistant Prof Bryan Bryson from the Massachusetts Institute of Technology. Bryan spoke about using cutting-edge single cell RNA sequencing technology to dissect activation states of macrophages infected with Mtb. He identified key regulatory proteins that are differentially expressed in granulocyte-macrophage colony-stimulating factor (GM-CSF) versus M-CSF differentiated macrophages and how these heterogenous macrophages play a role in the control of Mtb infection ^27^. He also described a new method they have developed to summarize transcriptomic heterogeneity within a dataset using geometric sketching ^28^. This method allows for greater understanding of macrophage heterogeneity and spatiotemporal localisation within granulomas. He also talked about phagosomics, a new way of measuring phagosome maturation and identifying new genes/proteins associated with phagosome formation during Mtb infection. He also talked about how to build a phagosome *de novo*, which enables for large scale testing of Mtb host stresses such as drugs. This uses tagged Mtb strains in combination with gene expression data to allow for better understanding of phagosome transcriptional changes in the presence of multiple Mtb stresses.

## Way forward

Prof Valerie Mizrahi, the director of the IDM gave the closing speech at the end of the symposium. In her concluding remarks she said, “It’s with incredible passion that people are progressing TB research, and that is because we’re living with it. It’s incumbent on all of us to think about why we’re doing what we’re doing and to remember that at the end of the day, it’s about the TB patients. Ultimately, one of the things we want to do is put ourselves out of business.”

## Conclusion

It is evident from the talks given by the various speakers that important research is being done to combat TB at the IDM in the Faculty of Health Sciences at the University of Cape Town. The research ranges from the identification of new candidate drug targets, investigating the utility of repurposed drugs as host-directed drug therapies, understanding the transmission of the bacteria in high burden communities, investigating the immunology of TB/HIV co-infection and identification of biomarkers for TB disease progression. An important highlight of this year’s World TB Day Nanosymposium is that a bulk of this research is being undertaken and led by early career research; thus, demonstrating the depth and breadth of talented TB researchers at the IDM.

## Data availability

No data are associated with this article.
