# Supplementary material for: Intervening along the spectrum of tuberculosis: meeting report from the World TB Day nanosymposium in the Institute of Infectious Disease and Molecular Medicine at the University of Cape Town
Source: Gates Open Res. 2020 May 28;3:1491. Originally published 2019 Jun 12. [Version 4] doi: 10.12688/gatesopenres.13035.4 (PMC7241047; doi:10.12688/gatesopenres.13035.4)
Supplement: Peer review history

# Peer review: Reviewer response for version 3

**DOI:** 10.21956/gatesopenres.14231.r28220

**Published:** 2020-05-20

**Authors:** Graham H. Bothamley

**Affiliations:** 1 Department of Respiratory Medicine, Homerton University Hospital, London, UK; 2 Queen Mary University of London, London, UK; 3 London School of Hygiene and Tropical Medicine, London, UK

I think we will not make much more progress regarding the unanswered sections, but I hope the authors found the comments helpful when they write up manuscripts and theses.

 The remaining comments are details, apart from swapping references 2 and 3:

1. References 2 should be reference 3 and vice versa to make sense with the text.
2. Page 4, paragraph 5, line 15: “that pathogenic mycobacteria survive…” not “survives”.
3. Page 5, paragraph 5, line 4: “making TB the highest contributor” instead of “making the TB a highest contributor”.
4. Page 5, paragraph 5, line 11: “who are also starting…” instead of “who also are starting…”
5. Page 6, paragraph 3, line 2: Perhaps replace “a lot of research” with “important research”?

I do not need to see the revisions above.

Does the article adequately reference differing views and opinions?

No

Are all factual statements correct, and are statements and arguments made adequately supported by citations?

Partly

Is the Open Letter written in accessible language?

Partly

Where applicable, are recommendations and next steps explained clearly for others to follow?

No

Is the rationale for the Open Letter provided in sufficient detail?

Partly

Reviewer Expertise:

NA

I confirm that I have read this submission and believe that I have an appropriate level of expertise to confirm that it is of an acceptable scientific standard.

# Response

**Published:** 2020-05-21

**Authors:** Sabelo Hadebe

**Response to reviewer:**

The remaining comments are details, apart from swapping references 2 and 3: 

1. Reference 2 should be reference 3 and vice versa to make sense with the text.

***Responses:** We thank the reviewer for highlighting this oversight, we have changed references accordingly in the revised manuscript.*

2. Page 4, paragraph 5, line 15: “that pathogenic mycobacteria survive…” not “survives”.

***Responses: **We thank the reviewer for highlighting this oversight, we have changed survives to “survive” in the revised manuscript.*

3. Page 5, paragraph 5, line 4: “making TB the highest contributor” instead of “making the TB a highest contributor”.

***Responses:** We thank the reviewer for highlighting this oversight, we have changed “making TB a highest contributor” to “making TB the highest contributor” in the revised manuscript.*

4. Page 5, paragraph 5, line 11: “who are also starting…” instead of “who also are starting…”

***Responses: **We thank the reviewer for highlighting this oversight, we have changed “who also are starting…” to “who are also starting…” in the revised manuscript.*

5. Page 6, paragraph 3, line 2: Perhaps replace “a lot of research” with “important research”?

***Responses: **We thank the reviewer for the comment, we have changed “a lot of research" to “important research” in the revised manuscript.*

# Peer review: Reviewer response for version 2

**DOI:** 10.21956/gatesopenres.14175.r28064

**Published:** 2019-10-23

**Authors:** Graham H. Bothamley

**Affiliations:** 1 Department of Respiratory Medicine, Homerton University Hospital, London, UK; 2 Queen Mary University of London, London, UK; 3 London School of Hygiene and Tropical Medicine, London, UK

- This Open Letter describes a meeting held on World TB Day at the Institute of Infectious Diseases and Molecular Medicine at the University of Cape Town, South Africa.
- The abstract does not well describe the meeting. The material up to “The theme of the nanosymposium was…” could be deleted.
- A symposium consists of both presentations and discussion. The paper does not describe the discussion.
- The meeting took place in 2019. The WHO Global Report on Tuberculosis 2018 was published 6 months before the meeting and reflects data gained in 2017. The reference should therefore be corrected.  
- The statement in the abstract (which would be better in the preamble) that “In South Africa, 322,000 people were infected with TB in 2017” is actually the incidence of disease rather than infection and the wide confidence intervals (230,000-428,000) should be given.  
- Reference 2 is not a primary source estimate for either the prevalence of latent tuberculosis infection (see Behr *et al.*, 2018 ^1^) nor the prevalence of an immune response to tuberculin or in an interferon-gamma release assay (see Houben and Dodd, 2016 ^2^).  
- Although the WHO 2018 report records that there were 160,684 cases of MDR/RR-TB detected and notified in 2017, the same paragraph gives an estimated incidence of 558,000 cases. 
- The statement that “This underpins an urgent need to develop better and efficacious vaccines than the currently available BCG and to develop novel drugs for TB” misses several steps in logical argument from the previous sentences regarding epidemiology to this conclusion.  
- When describing the speakers’ contributions, it is helpful to present a brief statement indicating where their research fits into the TB research agenda. For instance, reference 3 indicates how tryptophan protects Mtb from macrophages stimulated by CD4+ T cells and reference 4 describes how arginine deprivation makes Mtb susceptible to killing via reactive oxygen intermediates. However, it is then unclear whether these essential amino acids were the “elements” sequestered by Mtb and were, in a different way, responsible for evading host recognition (not mentioned in either of the two references). How was riboflavin related to these references? My reading of the literature is that riboflavin metabolites might be recognized by MAIT cells.  
- The section on Dr Nakedi’s work is more understandable, although its positioning within New TB drugs rather than TB pathogenesis is difficult to fathom.  
- Under “Host-directed drug therapies”, statins should be mentioned in the first sentence and an explanation given as to how they might affect a successful response to respiratory pathogens, especially Mtb. The final sentence does not give the reasons why BATF2 and miR-143 and 365 might be relevant to the topic of host directed drug therapies.  
- The section on Dr Purihar’s work on barbarine is understandable.  
- The section on “Transmission of Mtb” appeared to deal with whole genome sequencing rather than transmission. It was not clear how these might be related. The need to obtain early samples would seem to relate more to the transcriptome (and proteome) of such bacilli and perhaps to epigenetic changes. 
- The section on IL-22 could usefully have referred to Ronacher *et al.* (2018 ^3^) as a summary of the position at the time of the symposium. New data (Ardain *et al.*, 2019 ^4^) make a more interesting role regarding group 3 innate lymphoid cells in mice in combination with IL-17.  
- The paragraph on the talk by Dr Shey would benefit from a listing of “predictive immunological biomarkers” which distinguish severe illness from likely death from tuberculosis.
- The paragraph “Engineering tools for TB” covers a range of topics and bullet points for the different parts of the talk would be helpful. The difference in the macrophage populations needs an introductory sentence. The term “parametric stitching” is a neologism – presumably sequences are aligned and differences noted?  
- This sounds an interesting symposium, but needs context and discussion to realise its value.  

Does the article adequately reference differing views and opinions?

No

Are all factual statements correct, and are statements and arguments made adequately supported by citations?

Partly

Is the Open Letter written in accessible language?

Partly

Where applicable, are recommendations and next steps explained clearly for others to follow?

No

Is the rationale for the Open Letter provided in sufficient detail?

Partly

Reviewer Expertise:

Tuberculosis

I confirm that I have read this submission and believe that I have an appropriate level of expertise to confirm that it is of an acceptable scientific standard, however I have significant reservations, as outlined above.

## References

[1] : Revisiting the timetable of tuberculosis. *BMJ*.2018; 10.1136/bmj.k2738 10.1136/bmj.k2738 PMC610593030139910

[2] : The Global Burden of Latent Tuberculosis Infection: A Re-estimation Using Mathematical Modelling. *PLoS Med*.2016;13(10) : 10.1371/journal.pmed.1002152 e1002152 10.1371/journal.pmed.1002152 27780211PMC5079585

[3] : IL-22: An Underestimated Player in Natural Resistance to Tuberculosis?. *Front Immunol*.2018;9: 10.3389/fimmu.2018.02209 2209 10.3389/fimmu.2018.02209 30319650PMC6167461

[4] : Group 3 innate lymphoid cells mediate early protective immunity against tuberculosis. *Nature*.2019;570(7762) : 10.1038/s41586-019-1276-2 528-532 10.1038/s41586-019-1276-2 31168092PMC6626542

# Response

**Published:** 2019-11-12

**Authors:** Sabelo Hadebe

**Response to reviewer**

·       This Open Letter describes a meeting held on World TB Day at the Institute of Infectious Diseases and Molecular Medicine at the University of Cape Town, South Africa.

·       The abstract does not well describe the meeting. The material up to “The theme of the nanosymposium was…” could be deleted.

Authors response: We note the comment from the reviewer. We have modified the abstract to address this comment by the reviewer.  

·       A symposium consists of both presentations and discussion. The paper does not describe the discussion.

Authors response: We note this comment by the reviewer. However, due to time constraints, we could not set aside time dedicated to discussion. However, there was an opportunity for engagement between speakers and guests during the poster session. Also, people were given time to ask question to speakers during the question and answer session. However, these questions and responses were not captured in this meeting report. We will look to implement this in future meeting reports. 

·       The meeting took place in 2019. The WHO Global Report on Tuberculosis 2018 was published 6 months before the meeting and reflects data gained in 2017. The reference should therefore be corrected.  

Authors response: We have corrected the reference.

·       The statement in the abstract (which would be better in the preamble) that “In South Africa, 322,000 people were infected with TB in 2017” is actually the incidence of disease rather than infection and the wide confidence intervals (230,000-428,000) should be given.  

Authors response: We have given the wide confidence interval as suggested by the reviewer.

·       Reference 2 is not a primary source estimate for either the prevalence of latent tuberculosis infection (see Behr *et al.*, 2018 ^1^) nor the prevalence of an immune response to tuberculin or in an interferon-gamma release assay (see Houben and Dodd, 2016 ^2^).  

Authors response: We thank the reviewer for bringing this to our attention. We have cited the review Behr et al., 2018 study by Houben and Dodd, 2019. 

·       Although the WHO 2018 report records that there were 160,684 cases of MDR/RR-TB detected and notified in 2017, the same paragraph gives an estimated incidence of 558,000 cases. 

Authors response: We have amended the sentence to reflect the estimated range by the WHO. The sentence now reads “In 2017, the WHO estimated that 558 000 (range 483,000-639,000) people developed resistance to rifampicin (RR-TB), and 82% of these people had multi-drug resistant TB (MDR-TB) ^1^”.

·       The statement that “This underpins an urgent need to develop better and efficacious vaccines than the currently available BCG and to develop novel drugs for TB” misses several steps in logical argument from the previous sentences regarding epidemiology to this conclusion.

Authors respone: The sentence is drawn from all the presented epidemiology presented in the paragraph. The aim of it is to chart a way forward in the fight to combat the scourge of TB.

·       When describing the speakers’ contributions, it is helpful to present a brief statement indicating where their research fits into the TB research agenda. For instance, reference 3 indicates how tryptophan protects Mtb from macrophages stimulated by CD4+ T cells and reference 4 describes how arginine deprivation makes Mtb susceptible to killing via reactive oxygen intermediates. However, it is then unclear whether these essential amino acids were the “elements” sequestered by Mtb and were, in a different way, responsible for evading host recognition (not mentioned in either of the two references). How was riboflavin related to these references? My reading of the literature is that riboflavin metabolites might be recognized by MAIT cells.  

Authors response: The two references show that Mtb requires some essential amino acids and if we disable its ability to sequester these amino acids, it becomes susceptible to immune mediated killing. Dr Chengalroyen described her research on nutrients required for Mtb survival and focus specifically on the metabolism of riboflavin and explored the draggability of this pathway. 

·       The section on Dr Nakedi’s work is more understandable, although its positioning within New TB drugs rather than TB pathogenesis is difficult to fathom.  

Authors response: We thank the reviewer for this comment. The candidate proteins identified by mass spectrometry can be investigated as potential candidate drug targets, thus, leading to the development of new drugs. We do however take the point of the reviewer but we think that the talk is still well placed in the topic of New TB drugs. 

·       Under “Host-directed drug therapies”, statins should be mentioned in the first sentence and an explanation given as to how they might affect a successful response to respiratory pathogens, especially Mtb. The final sentence does not give the reasons why BATF2 and miR-143 and 365 might be relevant to the topic of host directed drug therapies.  

Authors response: The statins are mentioned in the first sentence of the second paragraph and we provide a citation of the work published by Prof Guler and colleagues. The citation is provided for the readers who would like to know more about the role of statins in response to Mtb infection. The last sentence is stating the other molecular targets Prof Guler is investigating as HDT for TB. Citations are also provided for the readers that would want to know more about these targets and why they are important for TB.

·       The section on Dr Purihar’s work on barbarine is understandable.  

Authors response: Noted.

·       The section on “Transmission of Mtb” appeared to deal with whole genome sequencing rather than transmission. It was not clear how these might be related. The need to obtain early samples would seem to relate more to the transcriptome (and proteome) of such bacilli and perhaps to epigenetic changes. 

Authors response: Whole genome sequencing has been suggested as a tool to investigate and track transmission of Mtb (Hatherill et al., 2016. BMC Medicine), however, little work has been done in high burden settings to understand how genetically related Mtb strains change during transmission. The need to obtain early samples is not related to the transcriptome or proteome of such bacteria but related to capturing the strains that might be driving transmission in communities . 

·       The section on IL-22 could usefully have referred to Ronacher *et al.* (2018 ^3^) as a summary of the position at the time of the symposium. New data (Ardain *et al.*, 2019 ^4^) make a more interesting role regarding group 3 innate lymphoid cells in mice in combination with IL-17.  

Authors response: We thank the reviewer for bringing these references to our attention. We have cited Ronacher et al., 2018. 

·       The paragraph on the talk by Dr Shey would benefit from a listing of “predictive immunological biomarkers” which distinguish severe illness from likely death from tuberculosis.

Authors Response: We thank the reviewer for this comment. We have provided a list of predictive biomarkers and cited the recently published manuscript for readers with keen interest on the topic. 

·       The paragraph “Engineering tools for TB” covers a range of topics and bullet points for the different parts of the talk would be helpful. The difference in the macrophage populations needs an introductory sentence. The term “parametric stitching” is a neologism – presumably sequences are aligned and differences noted?  

Authors response: We have cited the recent article by Bryson et al.,2019 describing heterogenous macrophages and their contribution to the control of Mtb infection. We have also cited the article by Hie et al., 2019 that describes geometric sketching as a tool for summarizing single cell RNA sequencing data.

# Peer review: Reviewer response for version 1

**DOI:** 10.21956/gatesopenres.14148.r27372

**Published:** 2019-07-02

**Authors:** Larry S. Schlesinger

**Affiliations:** 1 Texas Biomedical Research Institute (Texas Biomed), San Antonio, TX, USA

This Open Letter is a nicely constructed report out of a meeting held in March 2019 around World TB day for the Institute of Infectious Disease and Molecular Medicine at the University of Cape Town in South Africa. The letter consists of a broad overview of relevant areas in tuberculosis (TB) research, brief summaries of the speakers at the meeting and a comment regarding the road forward. The program was by and large for local participants. However, there was participation from KwaZulu Natal, the University College London and MIT/Harvard.

 New information is provided that will be of interest to the field. However, the nature of this format necessitated that information was brief and not really detailed so that the reader only gets some insight into the general topics and way forward.

 My comments are mostly editorial:

- Page 3, first paragraph after the disclaimer: “Interestingly, almost one-third of the world population that is exposed to Mtb…control infection…latently infected.” Given current thoughts in the field, I would suggest changing the beginning of the sentence to something like: “Realizing the limitations of the TST and IGRA, it has been estimated (or it is assumed) that almost one-third…”.
- Page 3, third paragraph: “…mechanisms mycobacteria interferes with” should be “…mechanisms by which mycobacteria interfere with…”.
- Page 3, regarding the paragraph about PknG: Has it ever been proven that this enzyme enters the cytosol? It would be important to know regarding its presumed functions.
- The manuscript uses both tuberculosis and TB...should stick with TB once defined.
- Page 3, last sentence paragraph 5: “candidates targets” should be “candidate targets”.
- Page 3, paragraph 6: Is it repurposed drug or drugs? Also which drug is “this drug”?
- Page 4, first paragraph: “capturing Mtb such as respiratory….” Would add “the” after as.
- Page 4, third paragraph: “TB a highest contributor…” Would change “a” to “the”.
- Page 4, 5 ^th^ paragraph: “…after presenting to hospital…” Would add “the” after to.
- Page 4, 6 ^th^ paragraph: “allows” appears twice and should be followed by “for”.

Does the article adequately reference differing views and opinions?

Partly

Are all factual statements correct, and are statements and arguments made adequately supported by citations?

Yes

Is the Open Letter written in accessible language?

Yes

Where applicable, are recommendations and next steps explained clearly for others to follow?

Partly

Is the rationale for the Open Letter provided in sufficient detail?

Yes

Reviewer Expertise:

TB research, innate immunity, lung cellular immunity

I confirm that I have read this submission and believe that I have an appropriate level of expertise to confirm that it is of an acceptable scientific standard.

# Response

**Published:** 2019-07-22

**Authors:** Sabelo Hadebe

- Page 3, first paragraph after the disclaimer: “Interestingly, almost one-third of the world population that is exposed to Mtb…control infection…latently infected.” Given current thoughts in the field, I would suggest changing the beginning of the sentence to something like: “Realizing the limitations of the TST and IGRA, it has been estimated (or it is assumed) that almost one-third…”.

**Response:** Thank you for this comment, this is absolutely true, we have changed the sentence as suggested.

- Page 3, third paragraph: “…mechanisms mycobacteria interferes with” should be “…mechanisms by which mycobacteria interfere with…”.

**Response:** changed as suggested.

- Page 3, regarding the para graph about PknG: Has it ever been proven that this enzyme enters the cytosol? It would be important to know regarding its presumed functions.

**Response:** The enzyme is predicted to be secreted into the cytosol, but there is no biochemical proof that has definitely shown that it is in the cytosol.

- The manuscript uses both tuberculosis and TB...should stick with TB once defined.

**Response:** changed, Tuberculosis has been used once in Abstract followed by abbreviation TB in brackets, TB is then used throughout.

- Page 3, last sentence paragraph 5: “candidates targets” should be “candidate targets”.

- Page 3, paragraph 6: Is it repurposed drug or drugs? Also which drug is “this drug”?

**Response:** barberine, this has been added in main article now.

- Page 4, first paragraph: “capturing Mtb such as respiratory….” Would add “the” after as.

**Response:** changed as suggested.

- Page 4, third paragraph: “TB a highest contributor…” Would change “a” to “the”.

**Response:** changed as suggested

- Page 4, 5 ^th^ paragraph: “…after presenting to hospital…” Would add “the” after to.

**Response:** changed as suggested.

- Page 4, 6 ^th^ paragraph: “allows” appears twice and should be followed by “for”.

**Response:** changed as suggested.
